# Anterior inferior plating versus superior plating for clavicle fracture: a meta-analysis

**DOI:** 10.1186/s12891-017-1517-1

**Published:** 2017-04-18

**Authors:** Jie Ai, Shun-Li Kan, Hai-Liang Li, Hong Xu, Yang Liu, Guang-Zhi Ning, Shi-Qing Feng

**Affiliations:** 1 Tianjin Medical University, 22 Qixiangtai Road, Heping District, Tianjin 300070 China; 2 Department of Orthopaedics, Tianjin Medical University General Hospital, 154 Anshan Road, Heping District, Tianjin 300052 China; 3 Department of Orthopaedics, Yuci People’s Hospital, 262 Jingwei Road, Jinzhong, Shanxi Province 030600 China

**Keywords:** Anterior inferior plating, Superior plating, Clavicle fracture, Meta-analysis

## Abstract

**Background:**

The position of plate fixation for clavicle fracture remains controversial. Our objective was to perform a comprehensive review of the literature and quantify the surgical parameters and clinical indexes between the anterior inferior plating and superior plating for clavicle fracture.

**Methods:**

PubMed, EMBASE, and the Cochrane Library were searched for randomized and non-randomized studies that compared the anterior inferior plating with the superior plating for clavicle fracture. The relative risk or standardized mean difference with 95% confidence interval was calculated using either a fixed- or random-effects model.

**Results:**

Four randomized controlled trials and eight observational studies were identified to compare the surgical parameters and clinical indexes. For the surgical parameters, the anterior inferior plating group was better than the superior plating group in operation time and blood loss (*P* < 0.05). Furthermore, in terms of clinical indexes, the anterior inferior plating was superior to the superior plating in reducing the union time, and the two kinds of plate fixation methods were comparable in constant score, and the rate of infection, nonunion, and complications (*P* > 0.05).

**Conclusions:**

Based on the current evidence, the anterior inferior plating may reduce the blood loss, the operation and union time, but no differences were observed in constant score, and the rate of infection, nonunion, and complications between the two groups. Given that some of the studies have low quality, more randomized controlled trails with high quality should be conduct to further verify the findings.

**Electronic supplementary material:**

The online version of this article (doi:10.1186/s12891-017-1517-1) contains supplementary material, which is available to authorized users.

## Background

Clavicle fracture was one of the most common fractures, the overall incidence of it was 64 per 100,000 per year according to previous studies [1, 2]. Many different techniques were developed to treat it. The therapeutic methods for clavicle fracture have been well studied and the differences between non-operative management and operative management have been compared in many studies [3–5]. Furthermore, more and more evidences based on randomized controlled trials with high quality confirmed that non-operative intervention may increase the initial fracture displacement, the incidence of nonunion and the time to return to sports [6–8]. Hence, there was a growing appreciation that operative treatment could be more helpful than non-operative intervention for patients with clavicle fracture.

In addition, various methods can be used for clavicle fracture, and the plate and screw constructs were two most common methods [3]. Furthermore, the anterior inferior plating and superior plating were two techniques which were used in plate fixation and some studies have been performed to compare the two different methods for clavicle fracture. Nevertheless, the positions of plate remained controversial. Zlowodzki and colleagues presented that superior plating was associated with more symptoms [5]. In addition, evidences indicated that the anterior inferior plating may reduce the risk of damaging the underlying neurovascular bundle and implant prominence [9–13]. However, Robertson and colleagues demonstrated that compared with the anterior inferior plating, the superior plating was preferred because of an advantage in fracture fixation, which could be seen in routine activity [13]. Furthermore, a previous studies reported that the superior plating had greater biomechanical stability [14].

Because of these contradictory results, we conducted this meta-analysis to provide an overview and quantitative estimate of the two different plating technologies for clavicle fracture based on the current evidences.

## Methods

The present meta-analysis was performed according to the recommendations of the Cochrane Handbook for Systematic Reviews of Interventions and was reported in compliance with the PRISMA (Preferred Reporting Items for Systematic Reviews and Meta-Analyses statement) guidelines [15, 16].

### Search strategy and study selection

PubMed, EMBASE, the Cochrane Library, Sciencedirect, CNKI and WanFang were searched by two researchers independently from inception to June 21, 2016. We combined MeSH terms with text words in the electronic search to identify the relevant literature. The search terms regarding to “clavicle fracture” were combined with terms related to “anterior inferior plating” and “superior plating”. In addition, we also manually examined the systematic reviews, meta-analyses, and the included articles for further studies. There was no language restriction. Based on the titles and abstracts, two investigators picked out the potential eligible studies. And then the full text of the remaining studies were reviewed for eligibility. Any disagreement was resolved by discussion or consulting a third reviewer.

### Eligibility criteria

1. Participants: Patients who had clavicle fracture and underwent anterior inferior plating or superior plating were considered eligible for the inclusion criteria.
2. Intervention and comparison: The group in which patients underwent anterior inferior plating for clavicle fracture was regarded as intervention group, and the control group enrolled patients with clavicle fracture who underwent superior plating. Patients who had clavicle fracture but didn’t receive anterior inferior plating or superior plating were excluded.
3. Outcomes: Blood loss and operation time were selected as surgical parameters, and we also extracted the union time, constant score, and the incidences of infection, nonunion and complications as clinical indexes.
4. Study design: Randomized controlled trials, cohort studies, and case-control studies that compared anterior inferior plating with superior plating in patients with clavicle fracture were considered qualified.

### Data extraction and outcome measures

Two reviewers independently extracted data from the eligible articles using a standard data extraction form. If any disagreements appeared, another reviewer was consulted. The following information was extracted from each included article:

Characteristics of each study: type of research, author name, year of publication, total number of patients, number of patients in intervention group and control group, duration of follow-up, and number of clavicle fracture of different sides.

Characteristics of patients: mean age and sex distribution.

Interventions: in intervention group, patients with clavicle fracture were treated by anterior inferior plating, and in control group, superior plating was chose.

Outcomes: surgical parameters (blood loss and operation time); clinical indexes (union time, constant score, infection, nonunion, and complications). We will choose the longest follow-up time as the measurement time point for all of the outcomes.

### Assessment of methodological quality

We evaluated the risk of bias of randomized controlled trails based on the Cochrane Handbook. The items which we assessed were showed as following: random sequence generation, allocation concealment, blinding of participants and personnel, blinding of outcome assessors, incomplete outcome data, selective outcome reporting and other bias (baseline balance and fund). According to evaluation, all of the randomized controlled trails were determined as low risk of bias, high risk of bias, or unclear risk of bias.

The Newcastle-Ottawa Scale (NOS) [17], which included three domains with eight items, was used to assess the methodological quality of cohort studies. Furthermore, a semi quantitative principle of star system with a maximum score of nine stars was used to evaluate the quality of articles. Four of nine stars represented the appropriate selection of exposure and non-exposure cohort participants, two of nine stars represented the comparability of cohort, and the assessment of outcomes and follow-up were represented by three stars. Five or more stars out of a total of nine stars was regarded as good quality.

### Statistical analysis

We used a descriptive analysis to describe the characteristics of studies included. Review Manager, version 5.3 was used to analysis the data. For dichotomous outcomes, we calculated relative risk (RR) with 95% confidence interval (CI), and for continuous outcomes, standardized mean difference (SMD) and 95% CI were calculated. We used I^2^ to estimate the heterogeneity among different studies [18]. If I^2^ was greater than 50%, significant heterogeneity was considered. When I^2^ exceeded 50%, a random-effects model was used. Otherwise, a fixed-effects model was used. Furthermore, we also conducted meta-regression analyses using Stata, version 12.0 (Stata Corp, College Station, TX) to explore the effect of mean age and duration of follow-up on different outcomes. The Egger’s linear regression test and funnel plots were used to examine the possibility of publication bias if more than ten studies were included [19]. P value of less than 0.05 denoted statistically significant difference.

## Results

### Study search

**Fig. 1:**
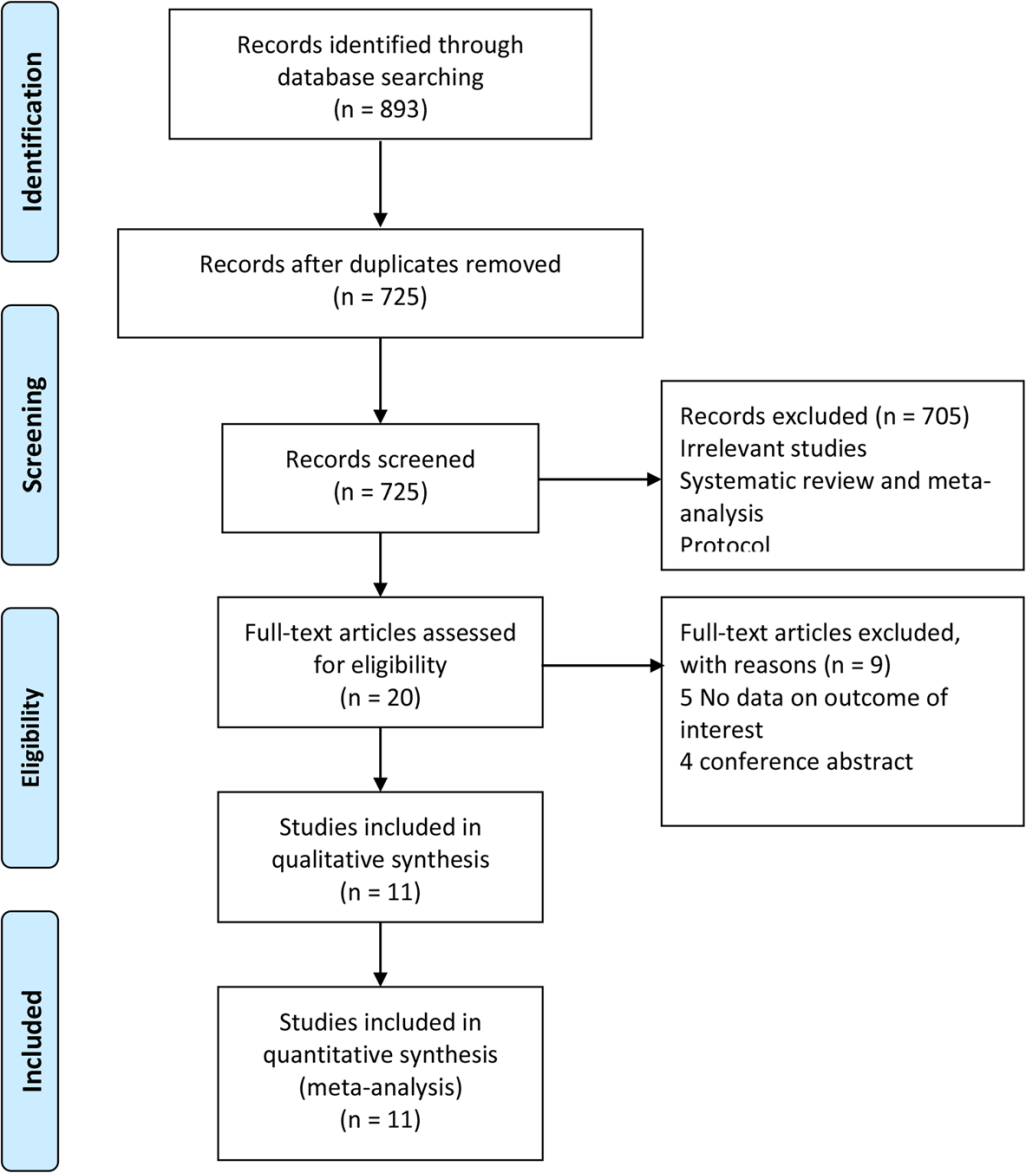
The flow diagram of study selection

Eight hundred ninety-three potentially relevant records were identified. One hundred sixty-eight records were ruled out due to duplicates. After screening the titles and abstracts, 34 records potentially fit our eligibility criteria. Then with full text screened, 12 studies were included [13, 20–30] in the final analysis. And the flowchart of literature selection are shown in Fig. 1.

### Study characteristics

Of the 12 articles included in our quantitative analysis, eight studies were retrospective studies [13, 21–24, 26, 29, 30], and the other four articles were randomized controlled trails [20, 25, 27, 28]. Only one study was multi-center study [13], and the rest of them were single-center study. Three studies included clavicle fracture of the aged [22, 25, 27], and midshaft clavicle fracture was studied in four studies [21, 23, 24, 26]. In addition, of the 12 articles, only one study reported the patient-reported symptoms and subjective outcome scores [26]. Finally, 954 patients (anterior inferior plating = 456, superior plating = 498) were included in our quantitative analysis. None of the studies received industry found. The information of study characteristics are presented in Table 1.

**Table 1:** Baseline characteristics of studies included in the meta-analysis

Source	Total no. of patients	Intervention	No. of patients	Follow-up, mo	Mean age, y	Fracture side			Gender	
						Left	Right	Bilateral	Male(%)	Female(%)
Cao 2015 [21]	42	Anterior inferior plating	23	-	46.4 ± 5.20	15	8	-	19	4
		Superior plating	19	-	48.5 ± 9.30	16	3	-	14	5
Qiu 2011 [28]	200	Anterior inferior plating	100	12	29	90	90	20	100	100
		Superior plating	100	12						
Deng 2008 [29]	67	Anterior inferior plating	29	6	28	54	50	-	59	50
		Superior plating	38	6						
Formaini 2013 [26]	105	Anterior inferior plating	43	2.00 ± 1.57	36.9 ± 13.2	21	22	-	27(63)	16(37)
		Superior plating	62	3.34 ± 2.01	36.1 ± 14.3	28	34	-	46(74)	16(26)
Hulsmans 2016 [13]	99	Anterior inferior plating	39	27 ± 19	38.6 ± 14.6	20	19	-	36(92)	3(8)
		Superior plating	60	21 ± 11	40.3 ± 11.5	29	31	-	55(92)	5(8)
Li 2013 [25]	66	Anterior inferior plating	33	6	68 ± 1.2	19	47	-	42	24
		Superior plating	33	6						
Qiu 2014 [22]	78	Anterior inferior plating	39	6.0	-	27	51	-	23	16
		Superior plating	39	6.0	-				24	15
Sohn 2015 [20]	37	Anterior inferior plating	18	16.7 ± 3.56	50.4 ± 17.36	7	11	-	18	1
		Superior plating	19	20.2 ± 9.85	46.7 ± 13.46	7	11	-	18	1
Xiao 2013 [23]	74	Anterior inferior plating	37	18	42.25 ± 2.92	17	20	-	19	18
		Superior plating	37	18	43.23 ± 3.16	18	19	-	20	17
Zhang 2012 [27]	92	Anterior inferior plating	46	6.0	67.9 ± 7.8	27	65	-	56	36
		Superior plating	46	6.0						
Zhao 2013 [24]	88	Anterior inferior plating	37	19.3	40.7	-	-	-	21	17
		Superior plating	51	19.3	42.5	-	-	-	38	13
Zheng 2006 [30]	25	Anterior inferior plating	13	16	36.5	-	-	-	16	9
		Superior plating	12	16						

### Methodological quality of the included studies

#### Risk of bias of randomized controlled trials

**Fig. 2:**
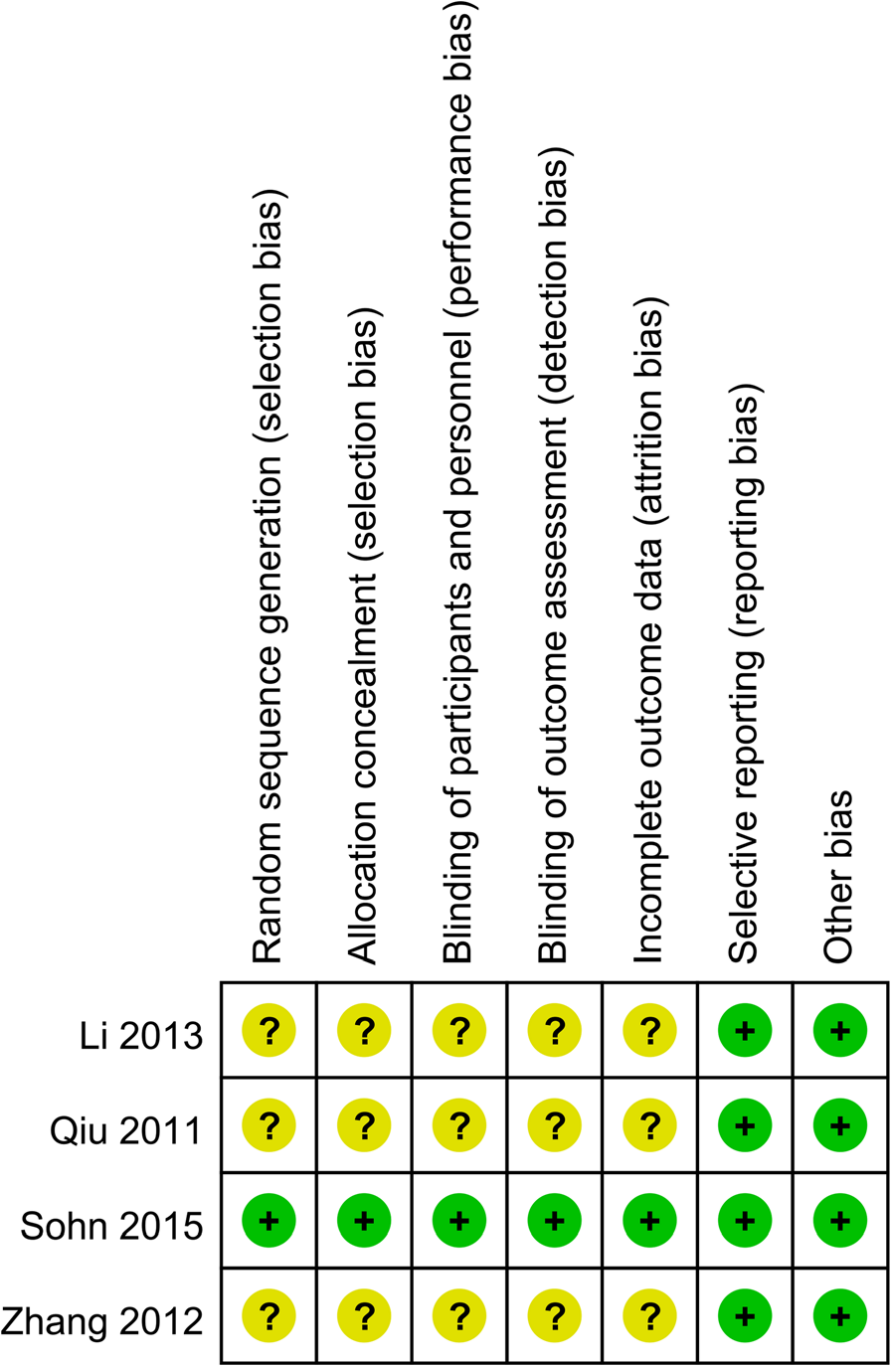
Risk of bias assessment of randomized controlled trials

There were four studies [20, 25, 27, 28] which were reported as randomized controlled trails. Only one study was regarded as low risk of bias [20], and other studies were considered to be at unclear risk of bias for the reasons of lacking enough information of random sequence generation, allocation concealment, blinding of the participants, and blinding of outcome assessment. In addition, only one study described the random sequence generation and allocation concealment detailedly [20]. Besides, none of the studies received foundation from any industries or other non-profit organizations. The outcomes of risk of bias of are presented in Fig. 2.

#### Methodological quality of cohort studies

There were eight studies which were reported as cohort study [20–24, 26, 29, 30]. All of them were considered as good quality. Five studies reported the ascertainment of exposure [13, 21–23, 26], and all of the eight studies laid down the period of follow-up appropriately before assessment. Furthermore, only two studies fully considered the comparability between different groups [13, 26]. The detailed information about risk of bias of these cohort studies are showed in Table 2.

**Table 2:** Methodological quality of cohort studies

Study	Selection				Comparability	Outcome			Score
	Representativeness of exposed cohort	Selection of non-exposed group	Ascertainment of exposure	Outcome of interest	Comparability of cohorts	Assessment of outcome	Length of follow-up	Adequacy of Follow-up	
Cao 2015 [21]	1	1	1	1	1	1	1	1	8
Deng 2008 [29]	1	1	0	0	1	1	1	1	6
Formaini 2013 [26]	1	1	1	1	2	1	1	1	9
Hulsmans 2016 [13]	1	1	1	1	2	1	1	1	9
Qiu 2014 [22]	1	1	1	1	1	1	1	1	8
Xiao 2013 [23]	1	1	1	1	1	1	1	1	8
Zhao 2013 [24]	1	1	0	0	1	1	1	1	6
Zheng 2006 [30]	0	1	0	0	1	1	1	1	5

### Surgical parameters

#### Operation time

**Fig. 3:**
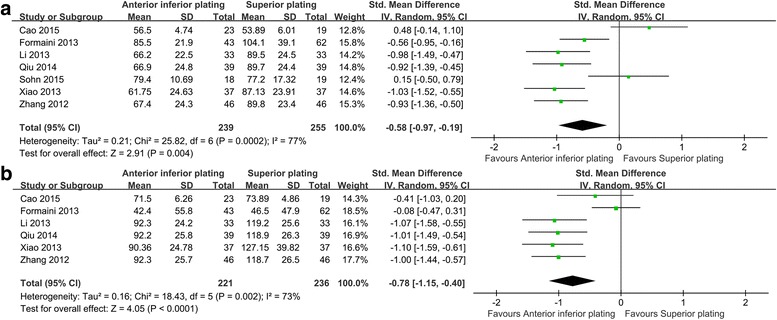
Comparison of operation time (**a**) and blood loss (**b**) in patients undergoing anterior inferior plating versus superior plating for clavicle fracture

There were seven studies (*n* = 475) provided available data for operation time [20–23, 25–27]. Of them, four studies were cohort studies [21, 22, 25, 27] and three studies were reported as randomized controlled trials [20, 23, 26]. The result of our meta-analysis showed that the operation time of anterior inferior plating was shorter than superior plating (SMD = −0.58, 95% CI −0.97 to −0.19, *P* = 0.004; I^2^ = 77%; Fig. 3a). In addition, meta-regression analysis was preformed to explore whether mean age had an effect on the operation time. The result showed that the mean age did not have an effect on it (*P* = 0.48, see Additional file 1: Figure S1).

#### Blood loss

Six studies reported the estimated blood loss during the operation [21–23, 25–27]. Among the six studies, four of them were retrospective studies [21–23, 26]. Most of them did not report the detailed information regarding fracture pattern, and only one study [26] reported the OTA classification. The result showed that blood loss in the experiment group was less than that in the control group (SMD = −0.78, 95% CI −1.15 to −0.40, *P* < 0.0001; I^2^ = 73%; Fig. 3b). Furthermore, the meta-regression analysis showed that no effect of mean age on blood loss (*P* = 0.22, see Additional file 1: Figure S2).

### Clinical indexes

#### Union time

Seven studies with 521 patients reported the union time [20, 22–27]. Three of them [20, 25, 27] were randomized controlled trials, but only one study [20] reported the detailed information of time schedule for follow up and demonstrated that there was no difference in union time between the two groups (*P* > 0.05). However, in the other two randomized controlled studies, the results indicated that the anterior inferior plating had a significant advantage in union time over the superior plating (*P* < 0.05). Moreover, the other four studies were retrospective ones and showed that the anterior inferior plating was superior to the superior plating, but only one study showed statistically significant differences between the two groups.

#### Constant score

**Fig. 4:**
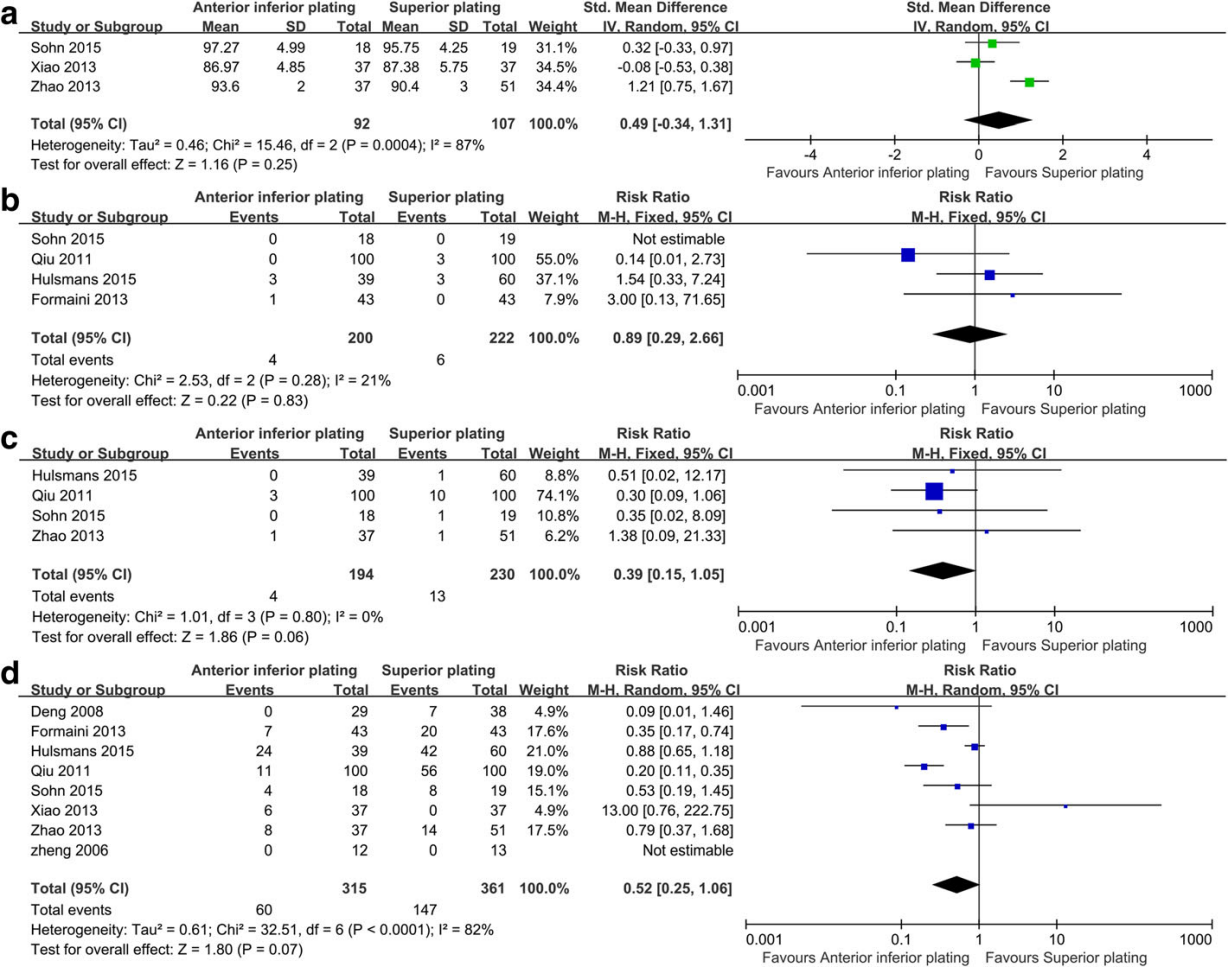
Comparison of constant score (**a**), infection (**b**), nonunion (**c**), and complications (**d**) in patients undergoing anterior inferior plating versus superior plating for clavicle fracture

Three studies with 199 patients [20, 23, 24] contributed to the analysis of constant score, and the constant score in the anterior inferior plating group was similar to that in the superior plating group (SMD = 0.49, 95% CI −0.34 to 1.31, *P* = 0.25; I^2^ = 87%; Fig. 4a).

#### Infection

There were four studies [13, 20, 22, 26] reporting the outcome of infection, and no significant difference were observed between two groups (RR = 0.89, 95% CI 0.29 to 2.66, *P* = 0.83; I^2^ = 21%; Fig. 4b).

#### Nonunion

The data regarding nonunion were available in four studies with 424 patients [13, 20, 24, 28]. No significant difference was found between the two groups (RR = 0.39, 95% CI 0.15 to 1.05, *P* = 0.06; I^2^ = 0%; Fig. 4c).

#### Complications

Eight studies with 675 patients reported complications [13, 20, 23, 24, 26, 28–30], such as infection, implant breakage, nonunion, screw pullout, irritation, and fracture deformity. Compared with the superior plating, the anterior inferior plating was not associated with a significant reduction in complications (RR = 0.52, 95% CI 0.25 to 1.06, *P* = 0.07; I^2^ = 82%; Fig. 4d). Furthermore, meta-regression analyses demonstrated that no relationship was observed between duration of follow-up and complications (*P* = 0.16, see Additional file 1: Figure S3), but the mean age affected the rate of complications (*P* = 0.02, see Additional file 1: Figure S4), i.e., trials with higher age presented higher rate of complications.

## Discussion

Plate fixation has been regarded as an effective treatment for clavicle fracture, which may result in a high rate of union and low rates of associated complications [31–33]. Anterior inferior plating and superior plating are two different plate fixation procedures [34]. As both of them have their certain advantages and disadvantages, which approach is more effective and safety is still in dispute. So we performed this meta-analysis to compare the effectiveness and safety between anterior inferior plating and superior plating for patients with clavicle fracture, and to our knowledge, this study was the first meta-analysis to fill this gap.

In this meta-analysis, we pooled the most recent evidence from both randomized and nonrandomized controlled trials, and provided the most reliable evidence. For surgical parameters, the results regarding operation time and blood loss demonstrated that the anterior inferior plating was better than the superior plating, which were consistent with Michael’s study [5]. This outcome may be explained by the following reasons. First, patients was supine during operation. When surgeon aligned the plate with the clavicle in an anteroinferior-to-posterior direction, the diameter of anterior inferior clavicle aspect was broader than the superior clavicle aspect, which may help surgeon operate smoothly and save much operation time. Second, in terms of plate bending, the anterior inferior plating could be more easily bent along the shape of the contralateral clavicle than the superior plating [20, 23]. However, four [21–23, 26] of those studies which reported the result of blood loss were retrospective review of the patients, and the method for calculating blood loss was based on the documentation recorded in the operation room, but not the reduced Hg after operation. And the other two randomized controlled studies [25, 27] also did not describe how they calculate the blood loss. Furthermore, all of them did not report the detailed information of fracture pattern which may also influence the blood loss. So this result may be biased.

For the clinical index of union time, there were seven studies reported this outcome [20, 22–27]. Four of them were retrospective studies [22–24, 26], and the other three studies [20, 25, 27] were randomized controlled trials. However, most of them did not report the detailed time schedule for follow up to evaluate the union time. Considering that different surgeons had different time schedule to evaluate the bone healing time, we could not merge the data to calculate the pooled effects. And all results showed that the anterior inferior plating had an advantage over the superior plating in reducing union time. The possible benefit of the anterior inferior plating is less skin irritation and less vascular compromise during the surgery. Furthermore, the placement of screws was away from the infraclavicular neurovascular structure [20], which may explain why the anterior inferior plating contributed to the union of clavicle fracture.

Besides, based on the pooled estimates, we found that the anterior inferior plating was similar with the superior plating in the rate of nonunion and complications. This was different from previous studies [35, 36]. In biomechanical studies, the superior plating presented superiority over the anterior inferior plating in fracture rigidity and the bending load to failure; however, the anterior inferior plating was superior in stability regarding bending rigidity [37, 38]. Both of them have their own advantages, so no differences were observed in nonunion and complications between two groups.

The strengths of this meta-analysis are presented as following. First, to the best of our knowledge, this is the first meta-analysis providing comprehensive insights into the comparison of the effectiveness and safety between the anterior inferior plating and the superior plating for clavicle fracture, which would provide a more reliable evidence for clinical practice. Second, we used strict retrieval conditions to identify the possible articles. Both randomized and non-randomized studies were included to provide sufficient sample size so that the inference is more reliable. Third, we also conducted meta-regression analyses to explore whether mean age and duration of follow-up were independent predictive factors for union time, operation time, blood loss, infection, nonunion and complications.

In addition, there were also several limitations which must be declared in this study. First, only 973 patients were included in this study. The number of patients was relatively small, which would limit the statistical power. Second, only four randomized controlled trials were included, and three of them were regarded as unclear risk of bias, which may influence the outcomes. Third, given that only five studies provided data for the number of each fracture side and the reasons of clavicle fracture, we could not conduct subgroup analyses to explore whether the fracture side or the reasons of clavicle fracture may influence the effect of surgery.

## Conclusions

Based on the current evidence, the anterior inferior plating may reduce the blood loss, the operation and union time, but no differences were observed in constant score, and the rate of infection, nonunion, and complications between the two groups. Given that some of the studies have low quality, more randomized controlled trails with high quality should be conduct to further verify the findings.

## Additional file

Additional file 1: Figure S1. Meta-regression analysis of influence of mean age on operation time. The circles represent each study. The size of the circle represents the power of the study. The solid line indicates the weighted regression line. **Figure S2.** Meta-regression analysis of influence of mean age on blood loss. The circles represent each study. The size of the circle represents the power of the study. The solid line indicates the weighted regression line. **Figure S3.** Meta-regression analysis of influence of mean age on union time. The circles represent each study. The size of the circle represents the power of the study. The solid line indicates the weighted regression line. **Figure S4.** Meta-regression analysis of influence of duration of follow-up on complications. The circles represent each study. The size of the circle represents the power of the study. The solid line indicates the weighted regression line. **Figure S5.** Meta-regression analysis of influence of mean age on complications. The circles represent each study. The size of the circle represents the power of the study. The solid line indicates the weighted regression line. (DOCX 257 kb)

## Abbreviations

CI: Confidence interval
NOS: Newcastle-Ottawa Scale
PRISMA: Preferred Reporting Items for Systematic Reviews and Meta-Analyses statement
RR: Risk ratio
SMD: Standardized mean difference
