# Supplementary material for: Anterior inferior plating versus superior plating for clavicle fracture: a meta-analysis
Source: BMC Musculoskelet Disord. 2017 Apr 18;18:159. doi: 10.1186/s12891-017-1517-1 (PMC5395806; doi:10.1186/s12891-017-1517-1)
Supplement: Additional file 1: Figure S1. — Meta-regression analysis of influence of mean age on operation time. The circles represent each study. The size of the circle represents the power of the study. The solid line indicates the weighted regression line. Figure S2. Meta-regression analysis of influence of mean age on blood loss. The circles represent each study. The size of the circle represents the power of the study. The solid line indicates the weighted regression line. Figure S3. Meta-regression analysis of influence of mean age on union time. The circles represent each study. The size of the circle represents the power of the study. The solid line indicates the weighted regression line. Figure S4. Meta-regression analysis of influence of duration of follow-up on complications. The circles represent each study. The size of the circle represents the power of the study. The solid line indicates the weighted regression line. Figure S5. Meta-regression analysis of influence of mean age on complications. The circles represent each study. The size of the circle represents the power of the study. The solid line indicates the weighted regression line. (DOCX 257 kb) [file 12891_2017_1517_MOESM1_ESM.docx]

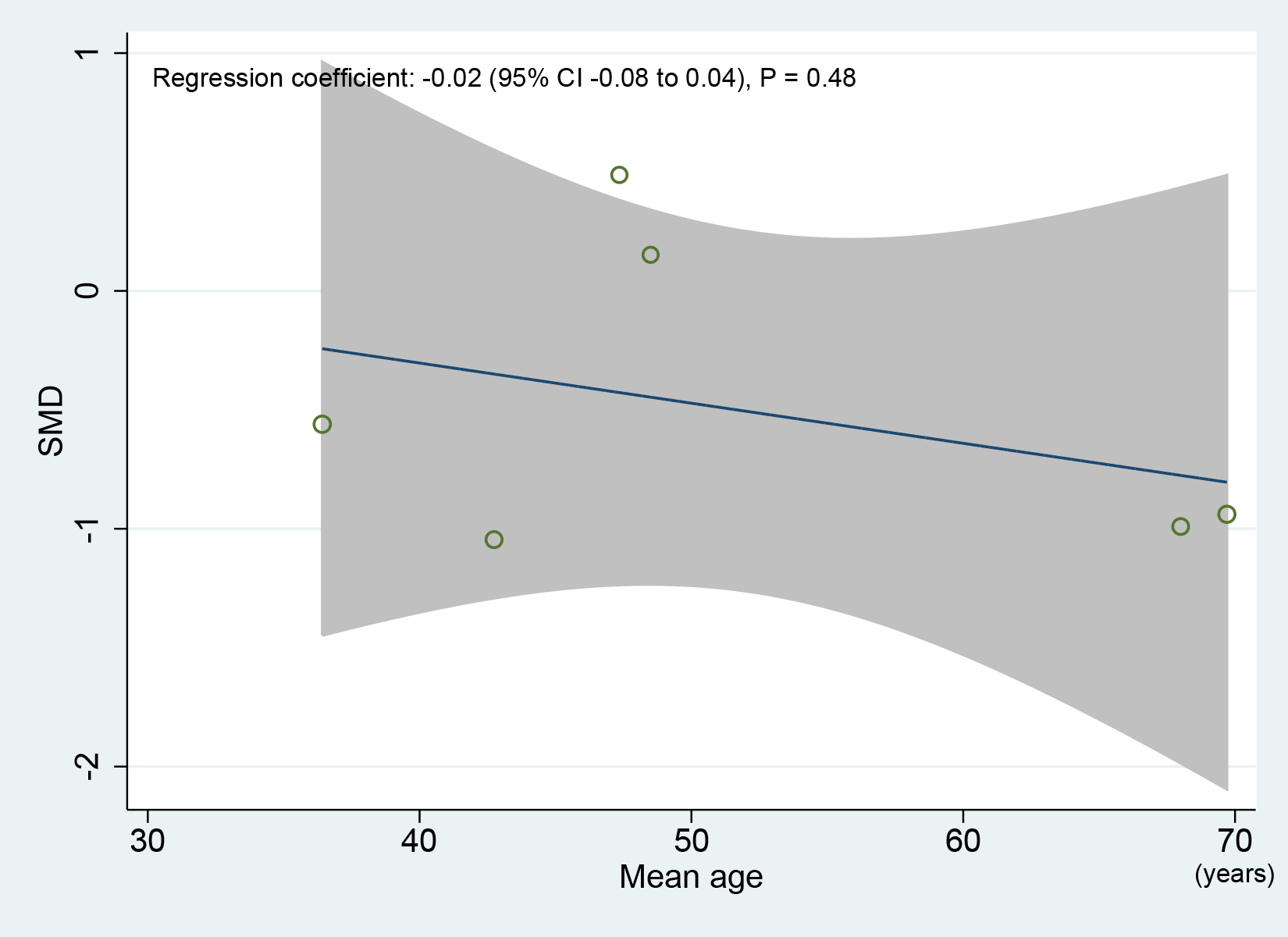


**Figure S1.** **Meta-regression analysis of influence of mean age on operation time.** The circles represent each study. The size of the circle represents the power of the study. The solid line indicates the weighted regression line.


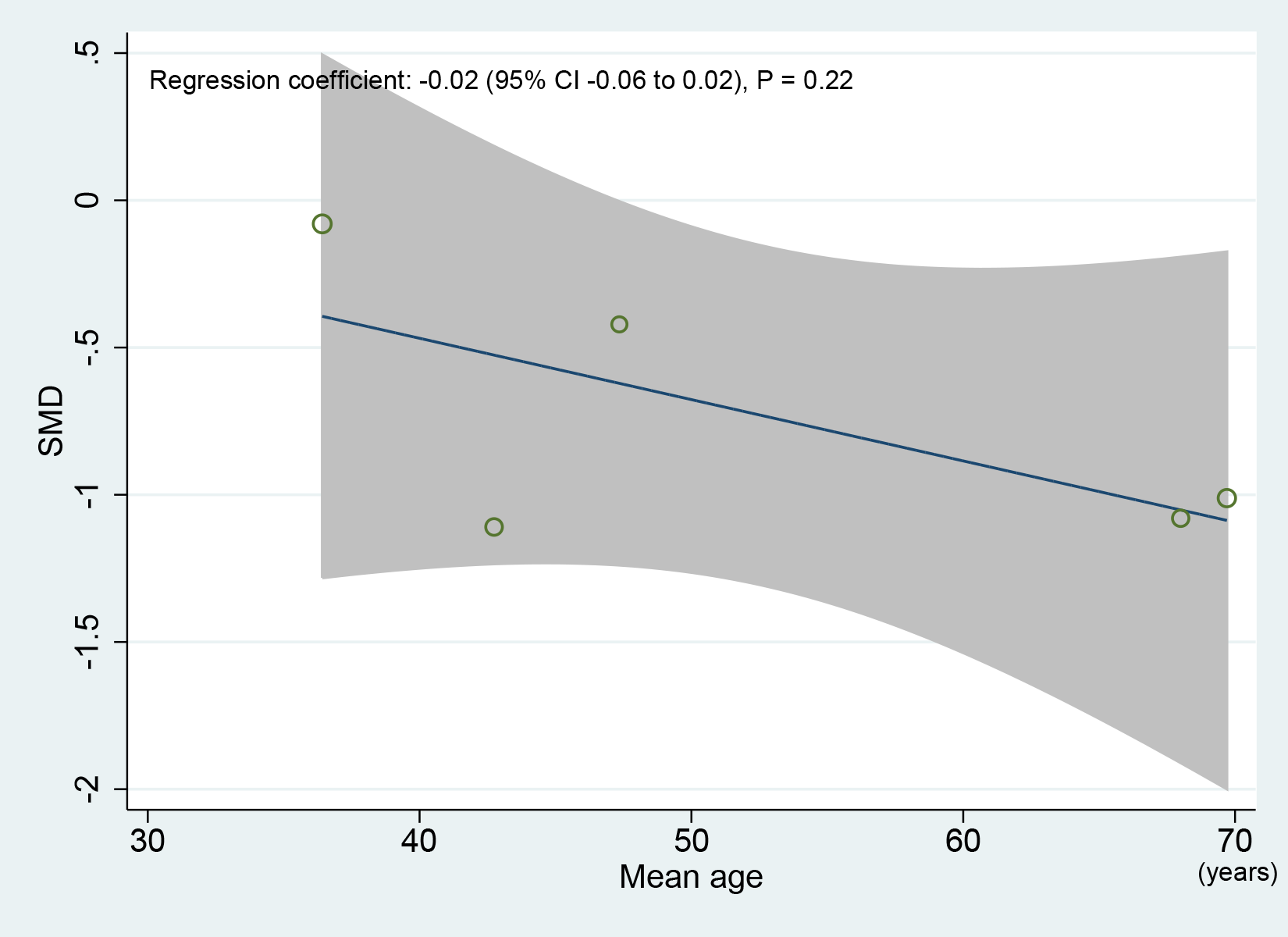


**Figure S2.** **Meta-regression analysis of influence of mean age on blood loss.** The circles represent each study. The size of the circle represents the power of the study. The solid line indicates the weighted regression line.


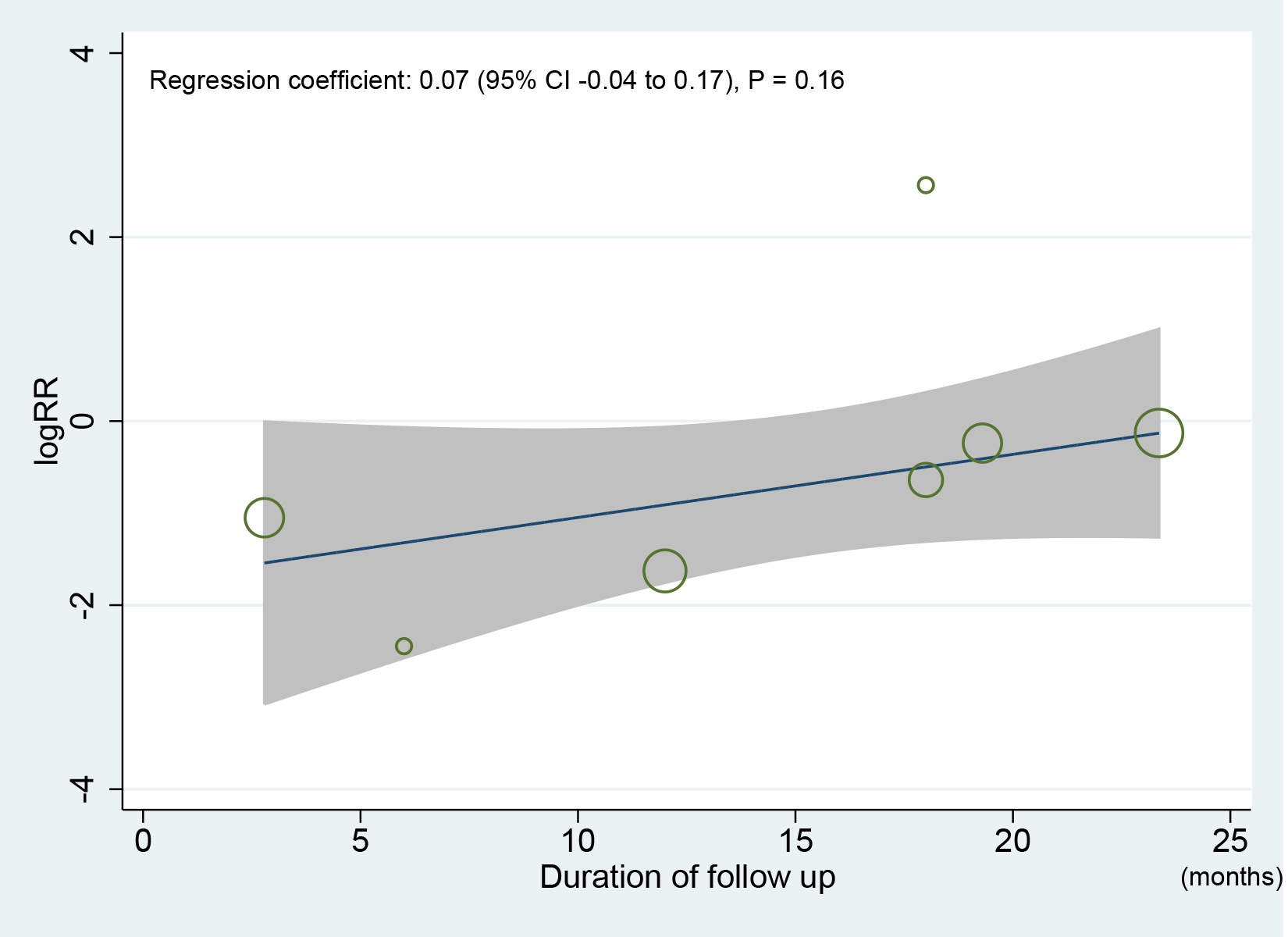


**Figure S3.** Meta-regression analysis of influence of duration of follow-up on complications. The circles represent each study. The size of the circle represents the power of the study. The solid line indicates the weighted regression line.


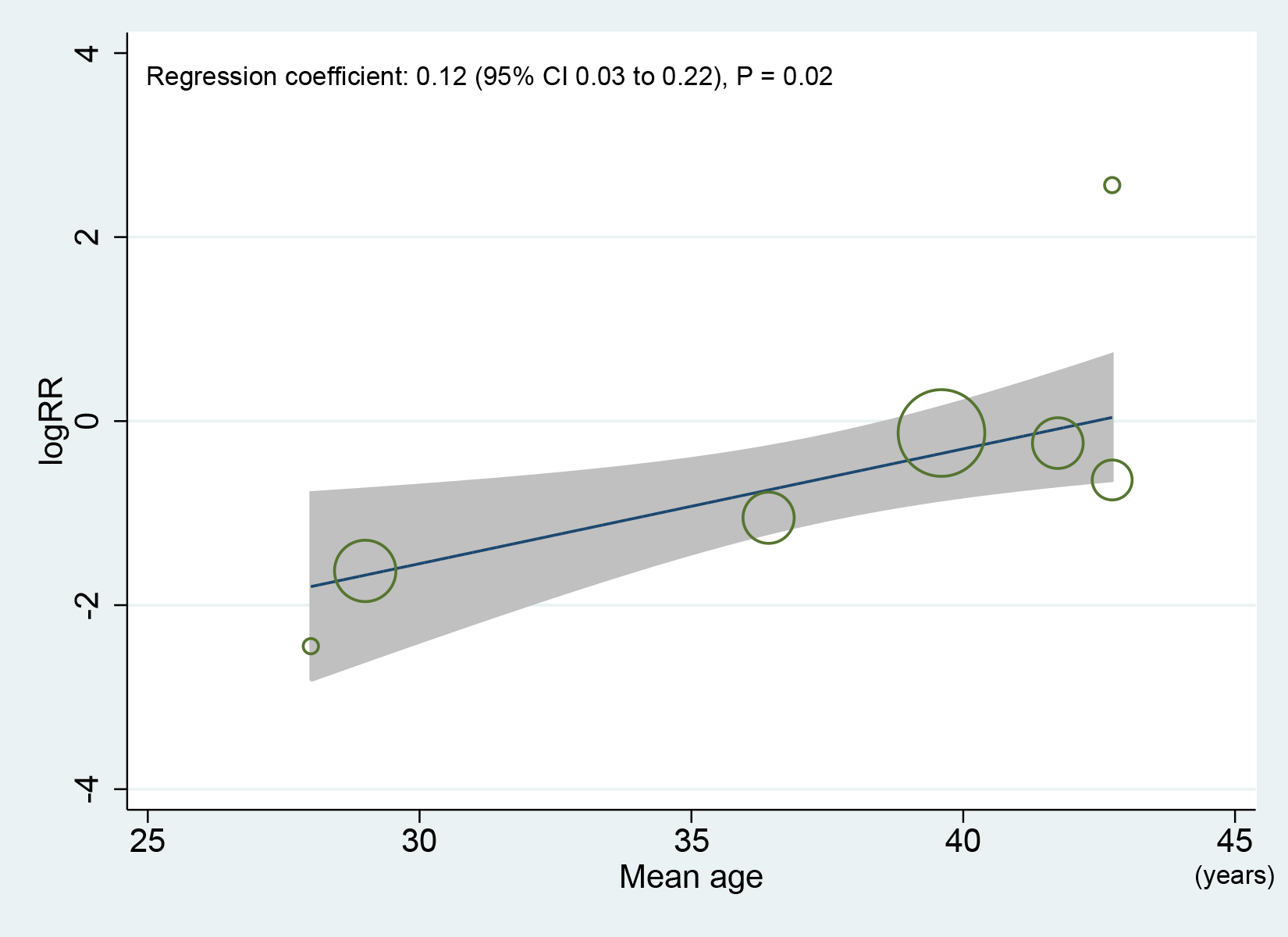


**Figure S4.** **Meta-regression analysis of influence of mean age on complications.** The circles represent each study. The size of the circle represents the power of the study. The solid line indicates the weighted regression line.
